# Evidence for trans-generational immune priming against *Vibrio splendidus* in the oyster *Crassostrea gigas*

**DOI:** 10.3389/fimmu.2025.1536562

**Published:** 2025-05-19

**Authors:** Xiaorui Song, Weilin Wang, Miren Dong, Chang Liu, Chuanyan Yang, Lingling Wang, Linsheng Song

**Affiliations:** ^1^ Liaoning Key Laboratory of Marine Animal Immunology and Disease Control, Dalian Ocean University, Dalian, China; ^2^ Jiangsu Province Engineering Research Center for Marine Bio-resources Sustainable Utilization, College of Oceanography, Hohai University, Nanjing, China; ^3^ Laboratory of Marine Fisheries Science and Food Production Process, Qingdao National Laboratory for Marine Science and Technology, Qingdao, China; ^4^ Dalian Key Laboratory of Aquatic Animal Disease Prevention and Control, Dalian Ocean University, Dalian, China

**Keywords:** trans-generational immune priming, *Crassostrea gigas*, transcriptomic analyses, DNA methyltransferases, E3 ligase

## Abstract

Cumulative evidence have demonstrated the occurrence of trans-generational immune priming (TGIP) in invertebrates; however, the detailed substances transferred, and the mechanism of this transmission remain unclear. In the present study, we first tested TGIP in the offspring of *Crassostrea gigas* after parental challenge with *Vibrio splendidus* during the spawning season. In the maternal oyster primed with *V. splendidus*, the enzyme activities (lysozyme and SOD), NO level, the expression of immune genes (*CgTLR2*, *CgMACPF*, and *CgFBG*), as well as the antibacterial activities were significantly enhanced in the eggs of *V. splendidus*-primed female oysters, indicating that *V. splendidus* stimulation promoted the immunity tendentiously transferred to eggs during the spawning season. After fertilization, the enzyme activities of CAT, lysozyme, and SOD were significantly enhanced in the maternal primed group [mVs-Sw (M)] during early oyster ontogeny, whereas there were no detectable differences between the control group (nSw-Sw (N)) and paternal primed group [pVs-Sw (P)]. However, the expression of immune genes (*CgGalectin*, Cg*Myd88*, and *CgLBP*) increased in the parental primed groups (mVs-Sw (M) and pVs-Sw (P)). After the larval offspring were exposed to the secondary *V. splendidus* stimulation, the mortality rates in the parental TGIP groups [mVs-Vs (M1) and pVs-Vs (P1)] were significantly lower, while the hatching rates were significantly higher than those in the nSw-Vs (N1), confirming that parents’ immunological experience enhanced their offspring survival rates as well as their resistance to pathogen infection. Transcriptome data revealed that differentially expressed genes were enriched in immunity, signal transduction, energy metabolism, and development in the parental TGIP groups. Notably, the expression levels of the three DNMTs were all significantly upregulated during early ontogeny in the maternal primed groups [mVs-Sw (M)], but sharply increased after entering the D-veliger larvae in the paternal primed group (pVs-Sw (P)), suggesting the potential regulation of DNA methylation during oyster TGIP. Moreover, the expression of E3 ligases (*CgWWP1*, *CgSmurf2*, *CgNedd4*, and *CgMarch5*) was significantly increased at the D-veliger and early umbo larval stages after *V. splendidus* stimulation, indicating their immune role during oyster ontogeny. These results are the first to show evidence of bacteria-induced TGIP and its potential mechanisms in mollusks.

## 1 Introduction

In contrast to B/T lymphocytes and antibody-mediated immune memory achieved by the acquired immune system, the innate immune system has also developed diverse mechanisms that “prime” for more robust and enhanced defense responses when encountering with the same pathogens, which are defined as trained immunity in vertebrates or immune priming in invertebrates (1, 2). Numerous reports have shown that trained immunity or immune priming can cross generations, which is termed transgenerational immune priming (TGIP) (3, 4). Although initially identified in vertebrates, increasing evidence corroborate that TGIP also occurs in invertebrates and is functionally equivalent to that in vertebrates (5–8).

The phenomenon of TGIP was first reported in the crustacean water flea *Daphnia magna* (8) and has so far been most extensively described in insect taxa (9), as well as a few other invertebrates (5). Currently, TGIP has been addressed in invertebrates, and priming effects have been proven to be triggered by the vertical and passive transfer of either active immune elicitors or information that establishes a primed state in the next generation, which provides a fitness advantage if offspring encounter a comparable immunological environment as their parents (9–11). However, routes to the realization of TGIP seem to be highly diverse among invertebrates (5, 9, 12–14). Most studies have demonstrated that parents pre-exposed to pathogens (bacteria, viruses, fungi, or metazoan parasites) show higher reproductive output, enhanced offspring survival rates, and resistance to pathogen infection (15–18). In addition, pathogen infection can induce specific transgenerational modifications in gene expression in offspring. For example, studies in the nematode *Caenorhabditis elegans* showed that the immune information transferred from the parents manifested as regulation of immune genes and/or protective aversion behaviors in the progeny (19). Transcriptome studies in insects have also shown that adults pre-exposed pathogens could improve the anti-parasitic and anti-bacterial defense of their offspring by priming the expression of immune-relevant genes (20–22). Further studies found that a restricted number of immunologically active proteins, peptides, or bacterial fragments were directly transferred from primed mothers to eggs. For example, global proteome analysis of the mealworm beetle *Tenebrio molitor* eggs from mothers primed with gram-positive and gram-negative bacteria showed an abundance of immunologically active proteins and peptides (14). In addition, induced levels of antibacterial activity, phenoloxidase (PO) activity, and antimicrobial activity were observed in the worker offspring of the bumblebee *Bombus terrestris* L. when their mother queen received a corresponding immune challenge prior to colony founding (18, 23–29). Similarly, maternal bacterial challenge led to a higher antibacterial defense of the oocytes produced in the marine annelid *Hediste diversicolor* (30). Trauer et al. showed that TGIP induced effects on PO activity, but not on antibacterial activity in offspring, and varied among different developmental stages in unchallenged offspring (31). Meanwhile, male bumblebees from immune-challenged groups showed increased constitutive immunity relative to the controls, which both enhanced encapsulation and protected against microorganisms (32, 33). Green et al. showed that oyster parents treated with poly(I:C) produced offspring with enhanced protection against ostreid herpesvirus type I infection and elevated levels of mRNA encoding a key transcription factor that regulates antiviral immunity (43).

However, to date, only a few underlying mechanisms of TGIP have been identified. First, it was proposed that TGIP appears to be mediated by the maternal transfer of bacteria or bacterial fragments to the developing eggs, as evidenced by tracing the fluorescent-labeled bacteria (34, 35). Moreover, Salmela et al. found that the egg yolk protein vitellogenin (Vg) can bind to bacteria and transport them into eggs, suggesting a central role for Vg in TGIP (36). Furthermore, Tetreau et al. deciphered the molecular mechanisms by using combined proteomic and transcriptomic approaches and revealed that antimicrobial peptides are specifically stored in eggs upon maternal bacterial priming (14). In recent years, a growing body of evidence suggests that epigenetic mechanisms may be responsible for TGIP (37–39). For instance, the acetylation and methylation levels of H4 and H3K4me3 histones in brine shrimp *Artemia stochastic* were detected in progenies whose ancestors were challenged, suggesting that epigenetic reprogramming of innate immune effectors is likely to play a central role in leading to trained immunity (40). Eggert et al. reported that paternal transgenerational immunity in the beetle *Tribolium castaneum* exposed to *Bacillus thuringiensis* is passed *via* sperm, and to a lesser degree seminal fluid, suggesting that epigenetic modifications involved the process (33). These studies concluded that TGIP was consistent for both maternal and paternal priming, and that the offspring might receive a double dose of protection. However, the underlying mechanisms require further investigation.

As filter-feeding mollusks in the intertidal zone, oysters are continuously exposed to pathogenic bacteria and viruses. There is increasing experimental evidence of immune priming in the oyster *Crassostrea gigas* (41, 42). During immune priming, modification of H3k4me3 enhanced the expression of *Cg*TLR3 in hemocytes to increase *Cg*IL17-1 production (41). Immune priming in oysters could also provide specific cross-protection against sympatric and allopatric *Vibrio splendidus* strains (42). Although evidence for TGIP in oysters has also been described in the offspring of mothers pretreated with poly I:C or OsHV-1 (43, 44), studies are yet to explicitly investigate the regulatory mechanism. In the present study, we aimed to investigate the mechanism of immunity transfer between maternal and offspring upon bacterial stimulation during the spawning season, and to examine and compare the immune changes in parental oysters of different genders during a transgenerational study. Furthermore, we aimed to unravel the mechanisms underlying the possible existence of TGIP by focusing on immunological pathways and epigenetic mechanisms.

## 2 Materials and methods

### 2.1 Ethics statement

All experimental procedures were conducted in accordance with the guidelines of the Care and Use of Laboratory Animals issued by the Ministry of Science and Technology in China. All oyster experiments were performed in accordance with animal ethics guidelines approved by the Ethics Committee of Dalian Ocean University.

### 2.2 Animals and bacterial strains

Gonadal-matured *C. gigas* oysters, with an average shell length of 13.0 cm ± 2 cm, were collected from a commercial shellfish farm (Changhai, Liaoning, China) during the spawning season. They were transferred from the farm to the hatchery for a two-weeks acclimatization period. Seawater was pumped directly from the sea of Heishijiao and filtered through a sand filter (40 μm) before draining the tanks. The oysters were fed Spirulina daily. To eliminate the bacteria carried out from the natural sea field, all oyster batches were subjected to a 5-day antibiotic treatment (10 mg/L of Flumequine, Shanghai Zhengyu Biotechnology Co., Ltd.) before subsequent experiments.

The pathogenic bacterial strain *V. splendidus* strain JZ6 was chosen to elicit a biparental immune response (45). A total of 100 μL of *V. splendidus* stored in 30% glycerol at −80°C, was inoculated into 100 ml 2216E media (tryptone, 5 g/L; yeast extract, 1 g/L; ferric phosphate 0.1 g/L; seawater, 1 L) at 16°C for 24 h. After centrifugation at 8,000×*g* at 4°C for 10 min, the bacterial pellets were washed three times and resuspended in filtered seawater to a final concentration of 2 × 10^8^ CFU/mL for subsequent experiments.

### 2.3 The immune priming stimulation of parents and *in vitro* fertilization

**Figure 1:**
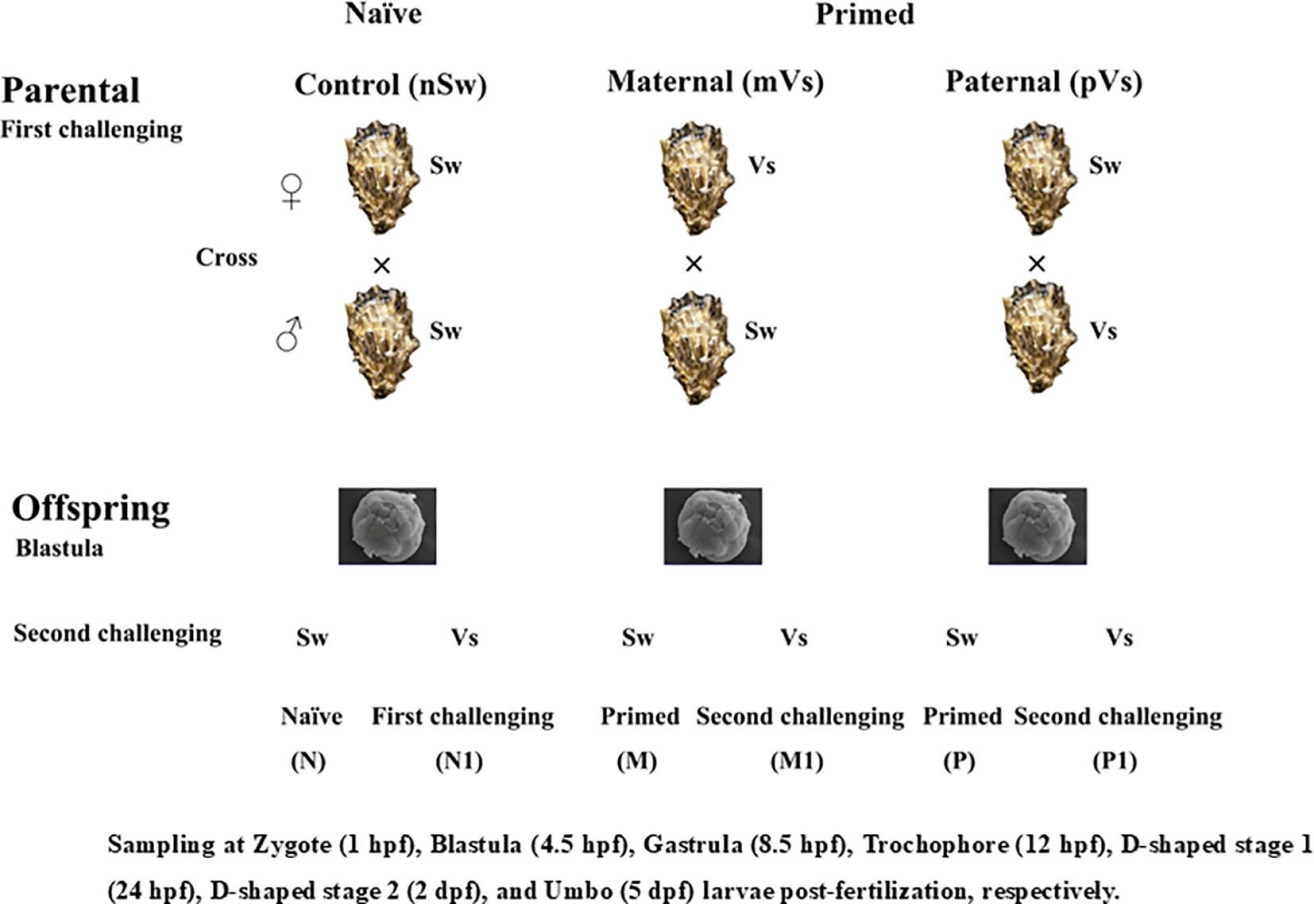
Graphical sketch of the whole experimental design. The parents were exposed to a priming with either bacterium (Vs: *Vibrio splendidus*), or equal sterile seawater (Sw). Breeding pairs with three different treatment combinations were formed and allowed to produce offspring. The sperm used in the ♀Vs ×♂Sw and ♀Sw ×♂Sw were from the same oysters; Similarly, the eggs used in the ♀Sw ×♂Vs and ♀Sw ×♂Sw were from the same oysters. in After developed into blastula stage, offspring larvae were re-exposed either to a bacteria challenge with Vs, or cultured in sterile seawater.

Two independent experiments were conducted (hereafter referred to as experiments 1 and 2), and the experimental design is shown in **Figure 1**. For experiment 1, gonadal matured oysters (160 individuals) were randomly divided into two groups and subjected to the following immune priming treatments: either injection of 100 μL *V. splendidus* (Vs, the Vs primed group) or equal sterile seawater (Sw, the naïve group). Hemocytes and eggs were collected by dissection at 1, 3, 5, and 7-days post-priming stimulation and directly stored in liquid nitrogen for enzyme activity assay or suspended in 1 mL TRIzol Reagent (Invitrogen, USA) for qualitative RT-PCR analysis.

*In vitro* fertilization was performed between different treatment groups for the convenience of synchronizing gametic activation and fertilization. Briefly, spawning and fertilization were performed artificially seven days post-priming (46). After *in vitro* dissection, eggs from the females of each treatment group (control Sw and Vs primed group) were dissected and rinsed together with filtered seawater into a 10 L bucket, while sperm from the males of each treatment group were rinsed in a beaker. The egg suspension was passed through a 90 μm nylon screen, rinsed on a 25 μm screen, and resuspended on 5 L of filtered seawater to a density of approximately 50,000 eggs/mL. Egg and sperm quality were checked *via* observation (bright orange color, cell integrity, and sperm motility) under an Inverted Metallurgical Microscope (Axio Observer A1, Zeiss, Germany). Qualified sperm were added to the egg suspension at a ratio of 1:10–1:15 based on the following pairing strategy: (1) both parents were naïve (control group, ♀Sw and ♂Sw), (2) *V. splendidus* primed female and naïve male (maternal primed group, ♀Vs and ♂Sw), (3) naïve female and *V. splendidus* primed male (paternal primed group, ♀Sw and ♂Vs) (**Figure 1**). Thus, approximately 250 million zygotes were obtained and incubated in 25 m^3^ sand-filtered seawater at 26°C. Salinity was around 30.

### 2.4 The immune stimulation of offspring and larvae collection

Experiment 2 was conducted after the embryos had developed to the blastula stage (4.5 h post-fertilization (hpf)). Briefly, embryos from the naïve group (n ≈ 3,000,000 embryos per tank) were divided into two groups, which remained unchallenged (nSw-Sw group, namely N group) and exposed to a challenge with *V. splendidus* (1 × 10^7^ CFU/mL, nSw-Vs group, namely N1 group). Embryos from the maternal-primed group (mothers were challenged with *V. splendidus* before spawning, N ≈ 3000,000 embryos per tank) were also divided into two groups and again exposed to a challenge with either *V. splendidus* (1 × 10^7^ CFU/mL, mVs-Vs (M1) group), or remained unchallenged (mVs-Sw (M) group). Embryos from the paternal primed group (fathers were challenged with *V. splendidus* before fertilization, N ≈ 3000,000 embryos per tank) were treated using the same method and two groups, pVs-Vs (P1) and pVs-Sw (P), were obtained (**Figure 1**). Each group was maintained in a 10-L plastic bucket containing recirculating filtered seawater. Three replicates were used for each treatment. Larvae were collected at 6 h, 12 h, and 24 h after *V. splendidus* challenge, suspended in 1 mL TRIzol Reagent (Invitrogen, USA), and immediately stored in liquid nitrogen. Three parallel samples were pooled as one biological replicate, with three biological replicates for each time point.

In addition, larvae at different developmental stages, including zygote (1 hpf), blastula (4.5 hpf), gastrula (8.5 hpf), trochophore larvae (15 hpf), D-veliger stage 1 (24 hpf), D-veliger stage 2 (48 hpf), and early umbo larvae (120 hpf), were collected and directly stored in liquid nitrogen for enzyme activity assays or suspended in 1 mL of TRIzol Reagent for qualitative RT-PCR analysis. The experimental scheme is illustrated in **Figure 1**.

Embryos developed into D-larvae (24 hpf) at a density of 100 larvae/mL. The tank water was changed every two days from this development stage onwards. The larvae were fed *Chrysophyceae* once daily. Larval density was assessed by means of microscope counts for each batch to further limit competition: larval concentration was progressively reduced from 10 to 5 and 3 larvae/mL on days 1, 5, and 7 post-fertilization, respectively. Sampling was performed at various stages of the oyster life cycle during the course of the experiment (46). The sieves (33 μm and 45 μm) were washed after each use and changed regularly depending on the spat growth.

Approximately 2 × 10^6^ larvae of blastula (4.5 hpf), D-veliger larvae 1 (24 hpf), and early umbo (120 hpf) stages were cultivated in three replicates with or without *V. splendidus* (1 × 10^7^ CFU/L). The larvae were collected at 6 h, 12 h, and 24 h after *V. splendidus* stimulation, suspended in 1 mL TRIzol Reagent, and immediately stored in liquid nitrogen for further mRNA extraction and transcriptome sequencing. Three parallel samples were pooled as one biological replicate, and there were three biological replicates for each time point.

### 2.5 Mortality statistics and the analysis of hatching rate of larvae

The mortality and hatching rates of oyster larvae were distinguished by microscopy, according to a previous study (47), and calculated in samples of 100–200 individuals after the embryo developed into the D-veliger stage (24 hpf). For mortality statistics, the swimming capacity is generally used to measure larval viability. In addition, the cilium and velum of vibrant larvae would protrude the shell after being anesthetized, and thus, they were considered as the criteria to determine mortality. The hatching rate refers to the percentage of embryos reaching the D-larvae stage according to the initial number of oocytes used for fertilization. All larvae were collected from each tank by sieving through a 45 μm nylon mesh and then placed in a graduated beaker. Larvae were observed and counted under a binocular microscope (Olympus BX41, ×100). At this stage, larvae that were not D-shaped were considered to be abnormal. Larval samples were placed at a concentration of 50,000 individuals per liter of filtered seawater and fixed using 50 μL of 8% paraformaldehyde (Sangon, China). The percentage of abnormal D-shell larvae was scored for 3 × 100 individuals in each group. Abnormalities (D-larvae presenting mantle and/or shell abnormalities) were identified.

### 2.6 Transcriptome sequencing and bioinformatic analysis

Total mRNA was isolated from D-veliger larvae of six experimental groups, including [nSw-Sw (N), nSw-Vs (N1), mVs-Sw (M), mVs-Vs (M1), pVs-Sw (P), and pVs-Vs (P1)], using TRIzol reagent following the manufacturer’s instructions. Qualified mRNA was converted into cDNA and then sequenced using the Ion Torrent Proton sequencing platform according to previous reports (48, 49). Three biological transcriptome sequencing replicates were used for each case. All bioinformatic analyses were performed according to a previous study (47), and the differentially expressed genes (DEGs) were further subjected to gene ontology (GO) and Kyoto Encyclopedia of Genes and Genomes Ortholog database (KEGG2) analyses.

### 2.7 Preparation of egg extracts and embryo immunity assays

Frozen gametes, larval samples and hemocytes were melted on ice and homogenized in 200 μL pre-cold PBS by using an electric homogenizer (T10 basic, IKA). The homogenate supernatants were collected as total protein extracts by centrifugation at 12,000×*g* at 4°C for 30 min, and the protein concentration was determined and quantified using the bicinchoninic acid method (BCA, Beyotime Biotechnology, China). The supernatants were divided into several aliquots and stored at −20°C.

The antibacterial activities of egg extracts from different groups were measured by detecting the bacterial growth curves. Prior to the assays, *V. splendidus* was grown overnight to stationary phase at 37°C in 5 mL of LB broth. A 1/50 dilution of the overnight culture was suspended in 5 mL fresh LB broth and grown for an additional 5 h at 37°C to obtain a mid-log-phase culture. The bacterial suspension was centrifuged, washed with PBS, and resuspended in PBS (1 × 10^6^ CFU/mL). Then, 100 μL of bacterial culture was mixed with 200 ng or 500 ng of egg extract (isolated from control and maternal primed groups, respectively, dissolved in PBS) and incubated at room temperature for 2 h. PBS and LB liquid medium were used as negative controls. Then, 20 μL of the mixture was added to a 96-well microliter plate with 200 μL of LB medium, which was placed in a microplate reader (Biotek, USA) at 37°C with shaking, and OD 600 was measured every 2 h to detect the growth of *V. splendidus*. Each experiment was repeated for three times.

The activities of superoxide dismutase (SOD), catalase (CAT), lysozyme (LYZ), and nitric oxide (NO) were measured using water-soluble tetrazolium salt (WST-1) and visible light and turbidimetry methods, respectively (Nanjing Jiancheng Bioengineering Institute, China).

### 2.8 Quantitative real-time PCR analysis of differentially expressed genes

Total mRNA was extracted using TRIzol reagent according to the manufacturer’s protocol. The quality and quantity of mRNA were assessed by electrophoresis on a 1% agarose gel and by Nanodrop 2000 measurement, respectively. Reverse transcription was performed with 1 mg of total mRNAs by using the Prime Script RT reagent Kit with gDNA Eraser (Takara, China) with oligo dT and random primers. The cDNAs from larvae at different developmental stages were used as templates after dilution to 1:50. The qRT-PCR reaction mixture (10 mL) consisted of 2×SYBR Green PCR Master mix, 0.4 mM each of the forward and reverse primers, and 1 mL of template cDNA. PCR amplification was performed under the following conditions: 95°C for 5 min, followed by 40 cycles of 95°C for 10 s, and 60°C for 30 s. Dissociation curve analysis was performed to determine target specificity. The specific primers for target genes and RS18 gene were prepared (**Table 1**). The relative expression ratio of the target genes versus the RS18 gene was calculated using the comparative Ct method (2^△△Ct^ method), and all data were given in terms of relative mRNA expression.

**Table 1:** List of primers used in this study.

Primer name	Sequence (5’-3’)
rt-*TLR2*-F	TTGGTACTTGGAAGTGTAACGACG
rt-*TLR2*-R	GGTCCGTGTTATTTGGCATGTG
rt-*MACPF*-F	TTCGCTGGTTTGTTCAGTTGTATGA
rt- *MACPF* -R	CGCTTTAAGGTAAGAGGAGAGAGAGTG
rt-*FBG*-F	TTTGTATTGTTTGAGGCAGTTATCC
rt- *FBG* -R	ACAGTGCTCTTGTAGTGTTTCCATG
rt-galectin-F	AAACTGTGCCTTCCACTTTAACCCC
rt- galectin-R	TGCATCGTAATCCCTCTCTTCTTCG
rt-*myd88*-F	ATTCCGACGTACCTTCAACTTGTCA
rt- *myd88*-R	GGGTAAGTCCACCTCCTCCCTGTTT
rt*-LBP*-F	AACTTACCACAAGGGTGAGGTGAAT
rt- *LBP* -R	TAAAGAGGAAGTCGGACAACCAGAG
rt-*DNMT1*-F	CCGAGGAAGAGACGACGGAACC
rt- *DNMT1*-R	TCAATCGCCCAGCATGACTCAGCA
rt- *DNMT2*-F	AGGTATATATCCCAGCAGCCACCTGTC
rt- *DNMT2*-R	CCTCCTCCTCCTCTCCCTCACTCA
rt-DNMT3B-F	GAGTCTGCTACGGCTGCAAAGC
rt-DNMT3B-R	TCATCAGTTTTCGGCTTCCGTCTCAT
rt-*WWP1*-F	AGGGAGCTGGAGCTGATGTTGTGTG
rt- *WWP1*-R	TGACCTGTTTGGAGTTCCTCTGGTAATG
rt-Smurf2-F	ATAGAGTTTGACGGAGAGGTGGGGC
rt- *Smurf2*-R	TCTGAAGCGTGTAGTTATCAGTAGCGGA
rt-*Nedd4*-F	ATAGAGTTTGACGGAGAGGTGGGGC
rt- Nedd4-R	TCTGAAGCGTGTAGTTATCAGTAGCGGA
rt-March5-F	AGACAAATCACCCAGGCCCAACATA
rt- *March5*-R	TTACACAGCACAGCACCAACAGACG
rt-*RS18*-F	GCCATCAAGGGTATCGGTAGAC
rt-*RS18*-R	CTGCCTGTTAAGGAACCAGTCAG

### 2.9 Statistical analysis

The expression level in the control was regarded as 1.0; therefore, the expression ratio of the treatments was expressed in relation to the control. Significant differences in expression levels between the control and treatment groups were analyzed as previously described. The significance level was set at *p <*0.05.

## 3 Results

### 3.1 The enzyme activities and NO, glucose level in eggs and hemocytes post *V. splendidus* priming

**Figure 2:**
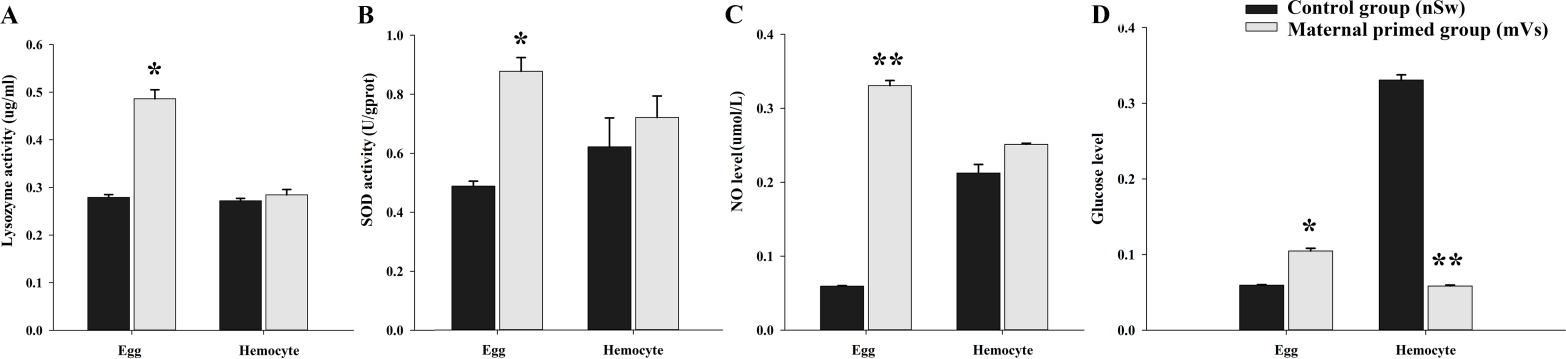
The enzyme activities and NO level in the eggs and hemocytes from control (nSw) and maternal primed (mVs) groups at 7-days post *V. splendidus* stimulation. **(A)** Lysozyme activity; **(B)** SOD activity; **(C)** NO level; **(D)** Glucose level; *p <0.05, **p <0.01.

To investigate the transfer of immune substances between mothers and offspring post-immune stimulation during the spawning season, the enzymatic activities of lysozyme and SOD, as well as NO and glucose levels, were examined in the eggs and hemocytes at 7-days post-priming from control (Sw) and Vs-primed females (experiment 1). In the eggs, compared with the control group (SW), the activities of lysozyme and SOD, and NO and glucose levels, all exhibited significant enhancement at 7-days post-priming stimulation (*p <*0.05, **Figure 2**). Briefly, the activity of lysozyme was 0.28 μg/mL in the control group (nSw) was 0.486 μg/mL in the maternally primed group (mVs), which was significantly upregulated (*p <*0.05, **Figure 2A**). Similarly, the activity of SOD was also significantly increased in the maternally primed group (mVs), and was 0.877 U/gprot, while there was only 0.487 U/gprot in the control group (nSw) (*p <*0.05, **Figure 2B**). For the NO production, compared with the control group (nSw), NO production was sharply increased in eggs from maternal primed group (mVs) at 7 days, which was 0.335 μmol/L in the maternal primed group (mVs), and only 0.073 μmol/L in the control group (nSw) (*p <*0.05, **Figure 2C**). There were no significant differences in lysozyme, SOD activity, or NO production in hemocytes at 7 days between the control group (nSw) and maternally primed group (mVs) (**Figures 2A–C**). The glucose level in the eggs from the maternally primed group (mVs) was slightly higher than that in the eggs from the control group (nSw) at 7 days (*p <*0.05, **Figure 2D**), whereas the glucose level in the hemocytes from the maternally primed group (mVs) was remarkably lower than that in the hemocytes from the control group (nSw) at 7 days (*p <*0.01, **Figure 2D**). These results suggest that female oysters specifically transfer and store antioxidant enzymes in eggs upon bacterial priming during the spawning season.

### 3.2 The expressions of immune-related genes in hemocytes and eggs post *V. splendidus* priming

**Figure 3:**
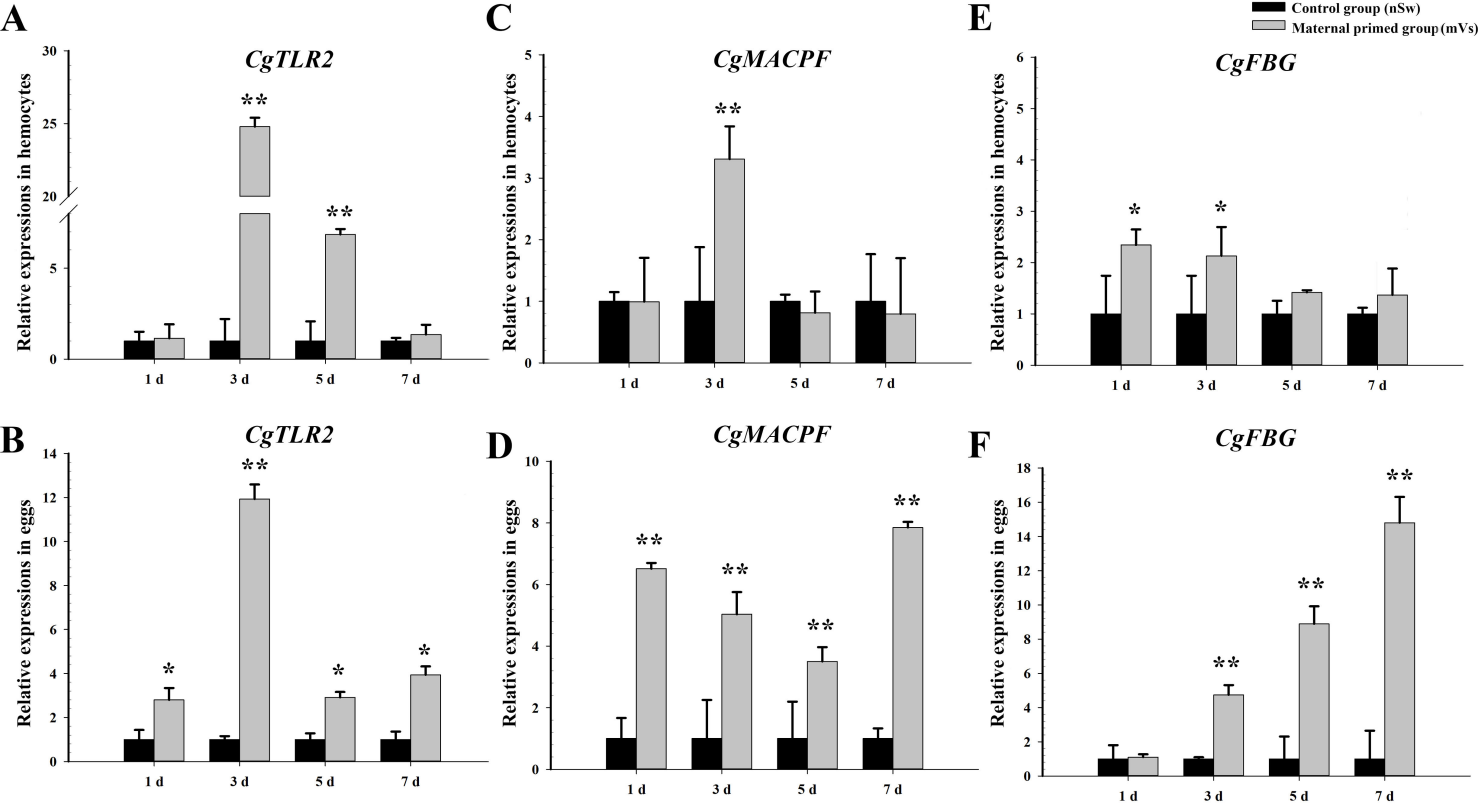
Expressions of immune-related genes in the hemocytes and eggs of oysters from control and maternal primed groups at different time points post *V. splendidus* stimulation. **(A, B)** The expressions of TLR2 in hemocytes and eggs, respectively; **(C, D)** The expressions of MACPF in hemocytes and eggs, respectively; **(E, F)** The expressions of FBG in hemocytes and eggs, respectively. *p <0.05, **p <0.01.

To investigate the transfer of immune substances between mothers and offspring after immune stimulation during the spawning season, the expression of immune-related genes (TLR2, MACPF, and FBG) was examined at different time points (1 day, 3 days, 5 days, and 7 days) in the eggs and hemocytes from the control group (nSw) and maternal primed group (mVs) (experiment 1). Compared with the control group (nSw), the mRNA transcripts of the three candidate genes were significantly increased in hemocytes and eggs from the maternally primed group (mVs) (**Figure 3**). Briefly, the transcripts of *CgTLR2* were only significantly upregulated at 3 days and 5 days in the hemocytes after bacterial priming (*p <*0.05, **Figure 3A**), while they exhibited a significant increase at all the tested time points (1 day, 3 days, 5 days, and 7 days) in the eggs, and showed the highest expression at 3 days in the maternally primed group (mVs), which was 11.93-fold of that in the control group (nSw) (*p <*0.05, **Figure 3B**). Similarly, the mRNA level of *CgMACPF* was upregulated only at 3 days in hemocytes after bacterial priming (*p <*0.01, **Figure 3C**). In contrast, the transcripts of *CgMACPF* were upregulated from days 1 to 7 in eggs after bacterial priming (*p <*0.01, **Figure 3D**). Compared with the control group (nSw), the expression of *CgFBG* was both significantly induced in the hemocytes and eggs from the maternally primed group (mVs) (*p <*0.05, **Figures 3E, F**). The expression of *CgFBG* in the hemocytes of the maternally primed group (mVs) was higher at 1 day and 3 days post-priming, which was 2.34- and 2.13-fold higher than that in the control group (nSw), respectively (*p <*0.05, **Figure 3E**). Notably, the transcripts of *CgFBG* in the eggs were upregulated from 3 days to 7 days post-bacterial priming and exhibited the highest expression at 7 days, which was 14.8-fold higher than that in the control group (nSw) (*p <*0.01, **Figure 3F**). These results collectively confirmed the transfer of immunity between mothers and offspring after immune stimulation during the spawning season.

### 3.3 Bacterial inhibition assay

**Figure 4:**
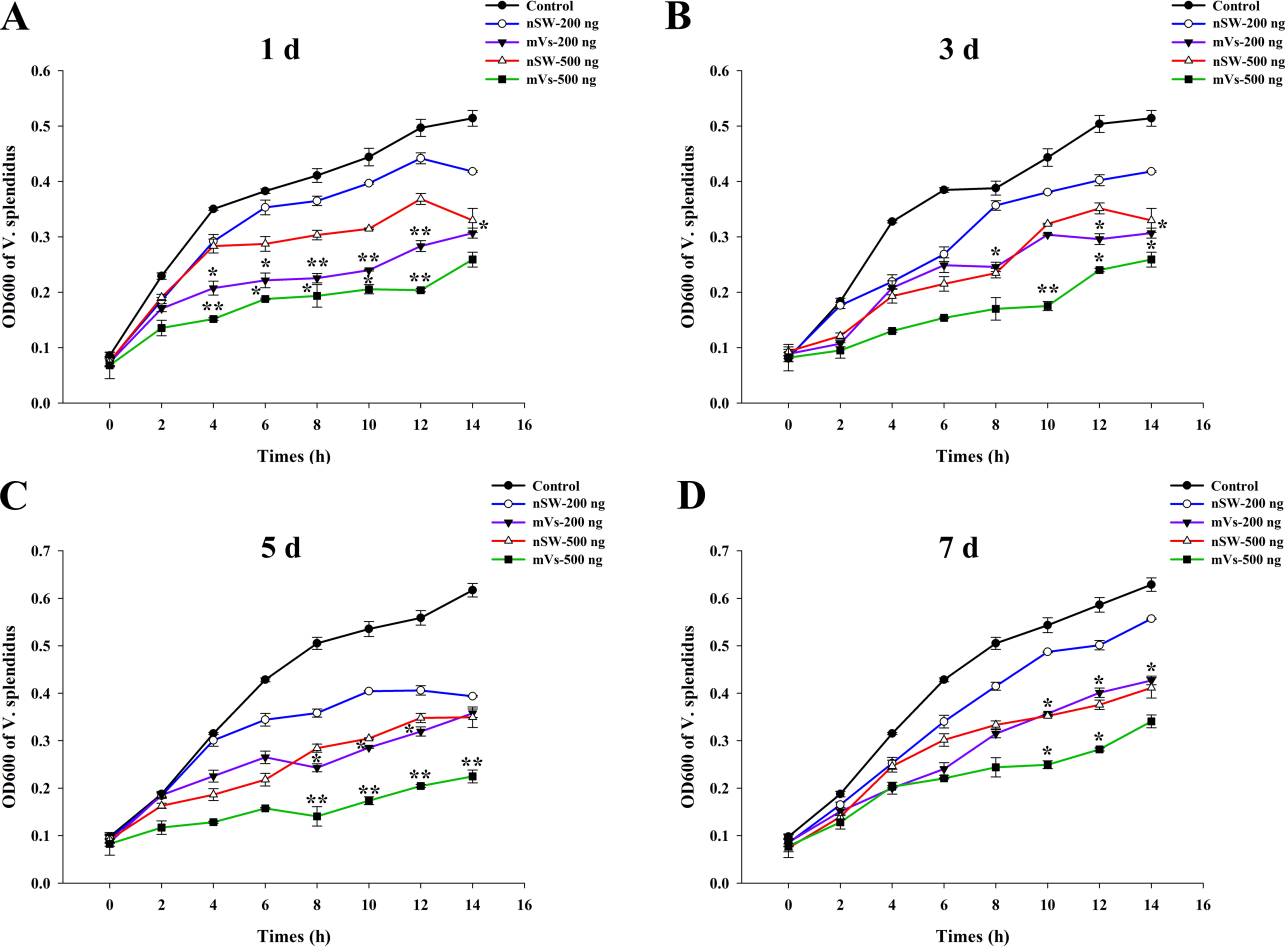
Antibacterial activity evaluation of egg extracts. The growth of *V. splendidus* was detected (the value of OD600) post the incubation with different concentrations (200 ng or 500 ng) of eggs extracts from different group (nSw, mVs) at different timepoints post *V. splendidus* stimulation. **(A)** 1 day; **(B)** 3 days; **(C)** 5 days; **(D)** 7 days; SW-200 ng: 200 ng egg extracts from control group (nSw); Vs-200 ng: 200 ng egg extracts from maternal primed group (mVs); SW-500 ng: 500 ng egg extracts from control group (nSw); Vs-500 ng: 500 ng egg extracts from maternal primed group (mVs); *p <0.05, **p <0.01.

To investigate the antibacterial activity of egg proteins extracted from control and *V. splendidus*-primed mature female oysters, a classical bacterial inhibition assay was performed. As shown in **Figure 4**, compared with the control group (PBS), egg extracts from either the control group (nSw) or maternal primed group (mVs) at all the time points (1 day, 3 days, 5 days, and 7 days) all showed inhibitory effects on the growth of *V. splendidus* in a dose-dependent manner (**Figures 4A–D**). In addition, compared with the egg extracts from the control group (nSw), equal amounts of egg extracts from the maternally primed group (mVs) at 1 day (**Figure 4A**), 3 days (**Figure 4B**), 5 days (**Figure 4C**), and 7 days (**Figure 4D**) all showed more remarkable antibacterial activities from 10 h to 14 h (*p <*0.05), confirming the induced anti-bacterial activity of eggs after *V. splendidus* priming.

### 3.4 The immunological activity during oyster early ontogeny

**Figure 5:**
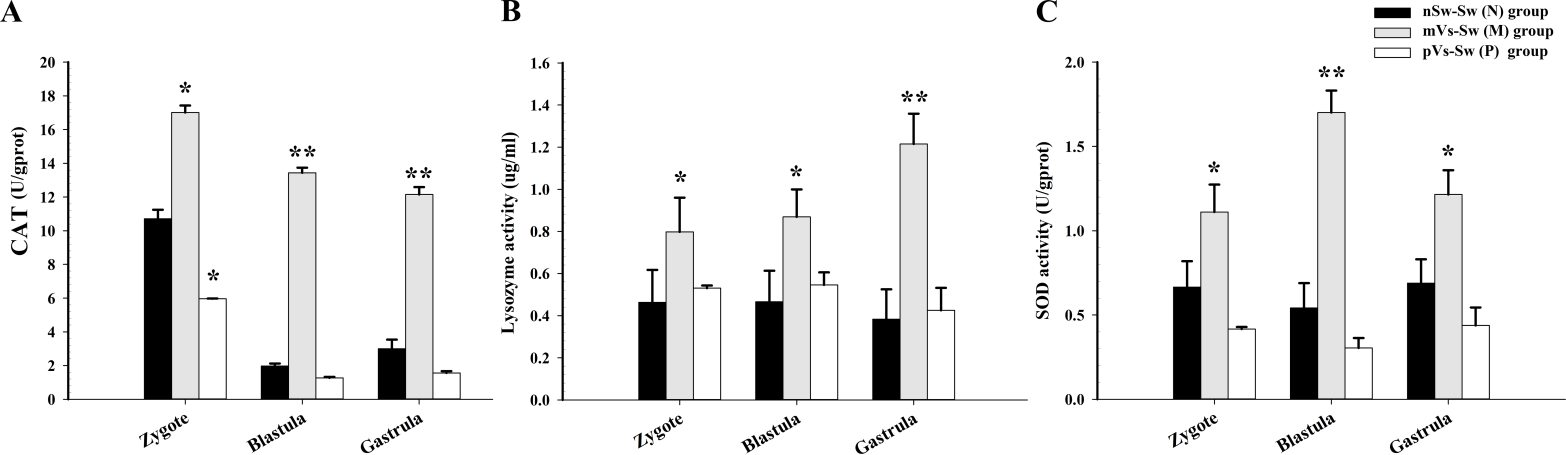
The enzyme activities during oyster early ontogeny in control, maternal primed and paternal primed groups. **(A)** CAT activity; **(B)** Lysozyme activity; **(C)** SOD activity; zygote (1 hpf), blastula (4.5 hpf), and gastrula (8.5 hpf). *p <0.05, **p <0.01.

To evaluate the effects of parental immune priming on larval oysters, the activities of two antioxidant enzymes, SOD and CAT, as well as lysozyme, were detected in the zygote (1 hpf), blastula (4.5 hpf), and gastrula (8.5 hpf) stages in the three groups [nSw-Sw (N), mVs-Sw (M), and pVs-Sw (P) groups in experiment 2, which were unchallenged with *V. splendidus* during ontogeny]. Briefly, compared with the control group nSw-Sw (N), the activities of the three investigated enzymes were significantly enhanced in the mVs-Sw (M) group (i.e., maternally primed group, *p <*0.05, **Figure 5**). In particular, the highest enzyme activities of SOD and CAT both appeared at the blastula stage (4.5 hpf) (*p <*0.01, **Figures 5A, C**), and the highest enzyme activities of lysozyme were detected at the gastrula stage (8.5 hpf) (*p <*0.01, **Figure 5B**). Compared with the control group, nSw-Sw (N), the activities of SOD and lysozyme showed almost no significant difference between nSw-Sw (N) and pVs-Sw (P) (paternal primed group) (**Figures 5B, C**). Additionally, the CAT activity in the pVs-Sw (P) group was lower than that in the control group (*p <*0.05, **Figure 5A**). These results collectively indicate that maternal immune priming induced immunological enzyme activity of the offspring during early ontogeny, while paternal immune priming showed little or no immunological effects on the larvae offspring.

### 3.5 The mRNA expression levels of immune-related genes during oyster early ontogeny

**Figure 6:**
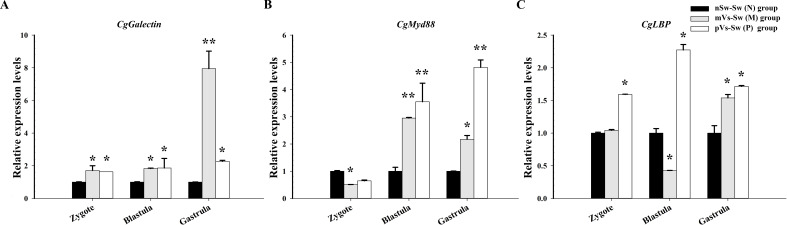
Expressions of immune-related genes during oyster early ontogeny in control, maternal primed and paternal primed groups. **(A)** galectin; **(B)** Myd88; **(C)** LBP; zygote (1 hpf), blastula (4.5 hpf), and gastrula (8.5 hpf). *p <0.05, **p <0.01.

To further investigate the effects of parental immune priming on the larval oyster, three immune-related genes (*Cg*galectin, *Cg*myd88, and *Cg*LBP) were selected based on the transcriptomic analysis, and the expression levels were quantified in the three groups [nSw-Sw (N), mVs-Sw (M), and pVs-Sw (P) groups in experiment 2]. For the *Cggalectin* gene, the relative expression of *Cg*galectin in the maternal immune priming [mVs-Sw (M)] and paternal immune priming [pVs-Sw (P)] was significantly upregulated at the zygote, blastula, and gastrula stages compared with the control group (nSw-Sw (N)) (*p <*0.05, **Figure 6A**). In particular, the expression level of *Cggalectin* at the gastrula stage in the mVs-Sw (M) group was 7.95-fold of that in the nSw-Sw (N) group (*p <*0.01, **Figure 6A**). For *Cgmyd88*, higher expression was observed at the blastula and gastrula stages in both the mVs-Sw (M) and pVs-Sw (P) groups (*p <*0.05, **Figure 6B**). In particular, the expression level of *Cgmyd88* at the blastula and gastrula stages in the paternal group was 3.6- and 4.8-fold higher than that in the nSw-Sw (N) group, respectively (*p <*0.01). In the mVs-Sw (M) group, the expression of *CgLBP* exhibited a slight upregulation at the gastrula stage compared to the nSw-Sw (N) group. The mRNA transcripts of *CgLBP* in the pVs-Sw (P) group were significantly upregulated at the zygote, blastula, and gastrula stages compared with those in the control group (*p <*0.05, **Figure 6C**).

### 3.6 Morphological characteristics of oyster larvae

**Figure 7:**
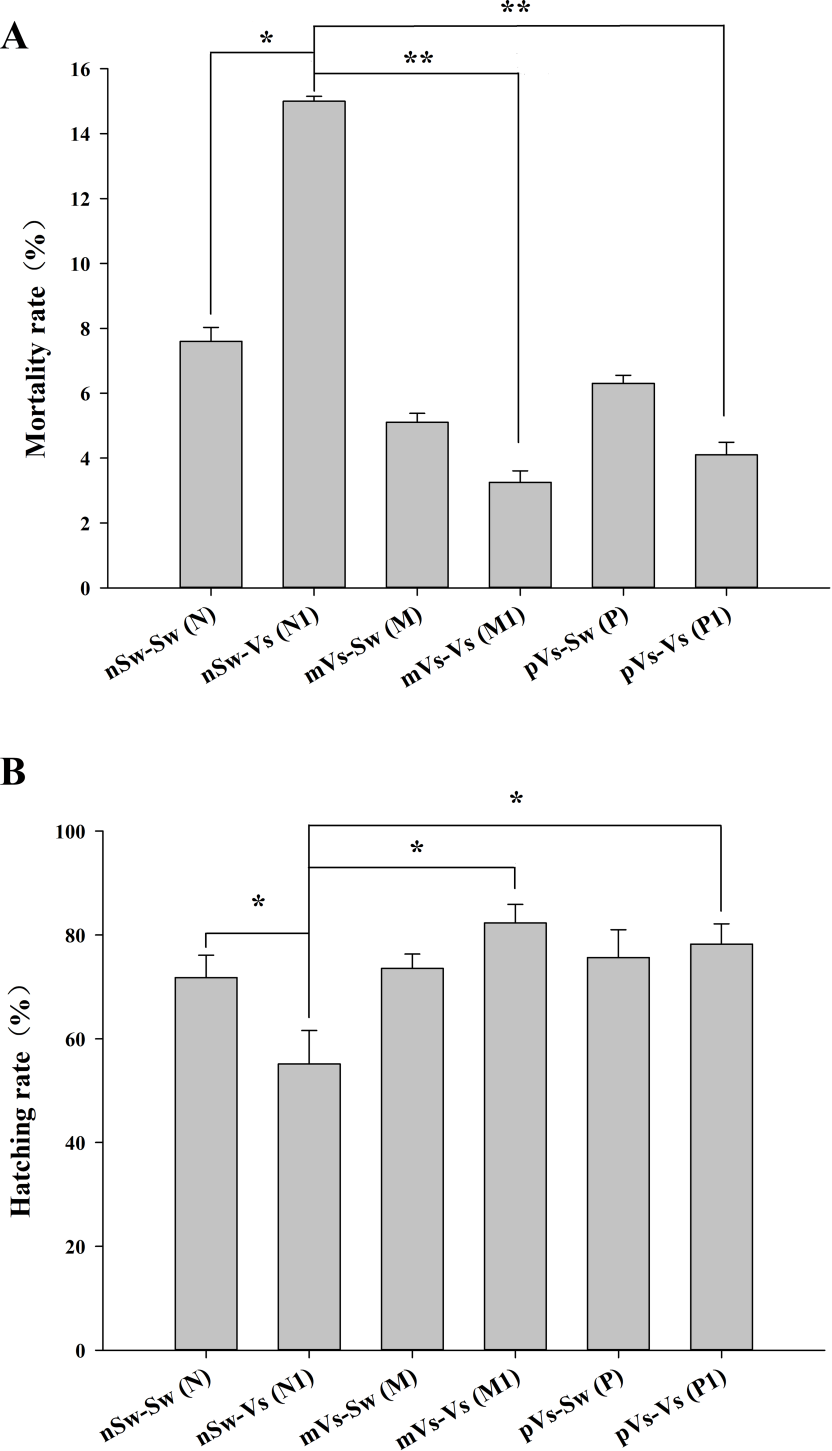
Mortality **(A)** and hatching rates **(B)** of D-veliger larvae oyster (24 hpf)) post-secondary *V. splendidus* stimulation in each group. nSw-Sw (N): both parental and blastula larvae oysters were unchallenged during the whole experiment; nSw-Vs (N1): parental oysters were unchallenged, but the blastula larvae were challenged with *V. splendidus*; mVs-Sw (M): maternal oysters were challenged with *V. splendidus*, but the blastula larvae were unchallenged; mVs-Vs (M1): both maternal and blastula larvae oysters were challenged with *V. splendidus*; pVs-Sw (P): paternal oysters were challenged with *V. splendidus*, but the blastula larvae were unchallenged; pVs-Vs (P1): both paternal and blastula larvae oysters were challenged with *V. splendidus*; *p <0.05, **p <0.01.

The performance of oyster larvae was observed using microscopy. For mortality statistics, the swimming capacity of D-veliger larvae and the position of cilium and velum after anesthetization were used to evaluate larval vitality. Based on observations and statistics, the mortality rate in the nSw-Vs (N1) group (parental oysters were normal, but larvae were bacterial challenged) was 15.3%, which was significantly higher (*p <*0.05) than that in the nSw-Sw (N) control group (parental and larval oysters were both normal) (7.6%, **Figure 7A**). The mortality rates in the parental-primed groups [mVs-Sw (M) and pVs-Sw (P)] (either parental oysters were bacterial challenged, but larvae were normal) were 5.1% and 6.3%, respectively (**Figure 7A**), both lower than those in the nSw-Sw (N) control group. Notably, the mortality rates were significantly reduced in the two TGIP groups [mVs-Vs (M1) and pVs-Vs (P1)] (parental and larval oysters were both bacterial challenged) compared to the nSw-Vs (N1) group (parental oysters were normal, but larvae were bacterial challenged), which were 3.25% and 4.1%, respectively (*p <*0.01, **Figure 7A**).

Hatching rate refers to the percentage of embryos reaching the D-larvae stage according to the initial number of oocytes used for fertilization. Based on the observations and statistics, it was 78.1% in the nSw-Sw (N) control group (parental and larval oysters were both normal). The hatching rate in the nSw-Vs (N1) group (parental oysters were normal, but larvae were bacterially challenged) (55.17%, *p <*0.05) was lower than that in the control group, which was consistent with the higher mortality rate in the nSw-Vs (N1) group (**Figure 7B**). Similarly, the hatching rates in the two parental primed groups (mVs-Sw (M) and pVs-Sw (P)) (either parental oysters were bacterially challenged, but larvae were normal) fit the low mortality rate and were slightly higher than that of the nSw-Sw (N) control group (**Figure 7B**). The hatching rates in the two TGIP groups [mVs-Vs (M1) and pVs-Vs (P1)] (parental and larval oysters were both bacterial challenged) were significantly higher than those in the nSw-Vs (N1) group (*p <*0.01), and the highest hatching rate (82.33%) was observed in the maternal TGIP group mVs-Vs (M1).

### 3.7 Identification of differentially expressed genes

D-veliger larvae (24 hpf) were sampled from six groups [nSw-Sw (N) and nSw-Vs (N1), mVs-Sw (M) and mVs-Vs (M1), pVs-Sw (P), and pVs-Vs (P1)], and RNA-Seq data were generated from 18 transcriptome libraries with three replicates in each group. A total of 28,027 genes were identified. By performing pairwise analysis of differentially expressed genes (DEGs) in each group, a total of 1,029 DEGs with a fold-change of gene FPKM value <0.5 or >2 were obtained (**Tables 2**. **3**). Among them, only 24 and 44 DEGs (upregulated) responded to the first stimulation of *V. splendidus* in female [nSw-Sw (N) and mVs-Sw (M)] and male [nSw-Sw (N) and pVs-Sw (P)] adult oysters 7 days before fertilization, respectively, and 479 DEGs responded to the secondary stimulation of *V. splendidus* that occurred at the blastula stage [nSw-Sw (N) and nSw-Vs (N1)], including 265 upregulated and 214 downregulated. Similarly, 321 DEGs were identified between the mVs-Sw (M) and mVs-Vs (M1) groups, including 190 upregulated and 131 downregulated genes, and 229 DEGs were identified between the pVs-Sw (P) and pVs-Vs (P1) groups, including 151 upregulated and 78 downregulated genes (**Tables 2**, **3**), these DEGs were mainly induced because of the secondary challenge at the blastula stage.

**Table 2:** Differentially expressed gene number of each group in maternal TGIP.

Comparison groups	Up	down	Total
nSw-Sw (N) & nSw-Vs (N1)	265	214	479
pVs-Sw (P) & pVs-Vs (P1)	151	78	229
nSw-Sw (N) & pVs-Sw (P)	44	0	44
nSw-Vs (N1) & pVs-Vs (P1)	196	242	438

**Table 3:** Differentially expressed gene number of each group in immune priming.

Comparison groups	Up	down	Total
nSw-Sw (N) & nSw-Vs (N1)	265	214	479
mVs-Sw (M) & mVs-Vs (M1)	190	131	321
nSw-Sw (N) & mVs-Sw (M)	24	0	24
nSw-Vs (N1) & mVs-Vs (M1)	136	170	306

### 3.8 Analysis of the differentially expressed genes in the parental TGIP

**Figure 8:**
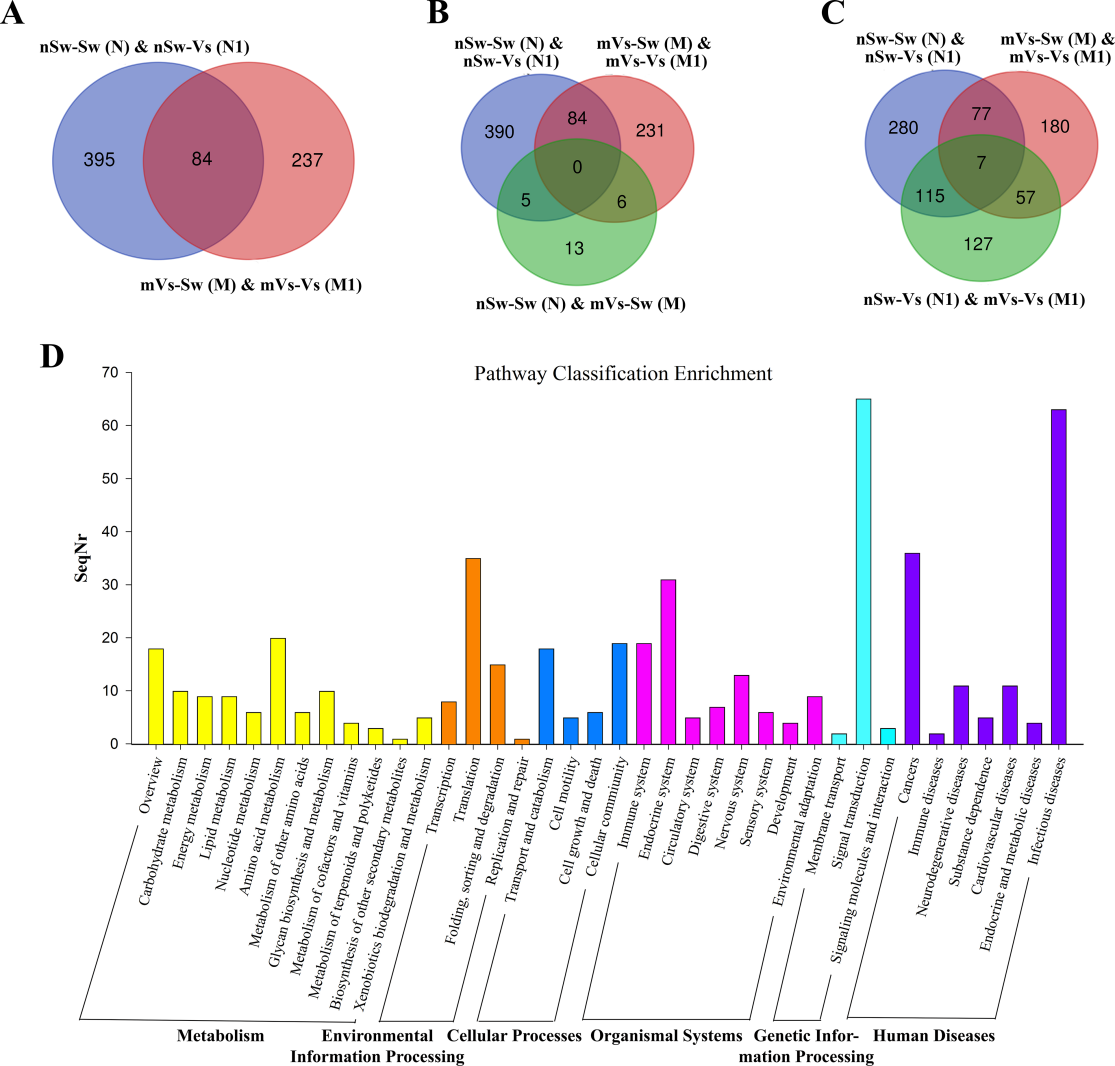
The analysis of differentially expressed genes in maternal primed larvae oysters. **(A)** The Venn diagram of differentially expressed genes in larvae oyster responding to both the first (against female oyster) and secondary stimulation of *V. splendidus*. **(B)** and **(C)** The Venn diagram of differentially expressed genes involved in maternal TGIP; **(D)** The KEGG enrichment of differentially expressed genes in oyster maternal TGIP.

First, to measure the ontogeny of larval innate immune system, the transcriptome of oyster D-veliger larvae in the present study [nSw-Sw (N)] was compared with the transcriptome of different tissues of oyster adults from previous reports (46, 50). More than 1,300 innate immune genes were identified in the transcriptome of nSw-Sw (N), and these genes were distributed in multiple immune signaling pathways, such as the Toll-like and TNF signaling pathways (**Supplementary Figure 1**). Second, to explore the potential molecular mechanism in the maternal TGIP, 84 DEGs were screened by comparing nSw-Sw (N) and nSw-Vs (N1) with mVs-Sw (M) and mVs-Vs (M1), which responded to the secondary bacterial stimulation occurring at the blastula stage (**Figure 8A**). These genes were further analyzed to explore DEGs between the unstimulated group [nSw-Sw (N)] and the maternally primed group mVs-Sw (M), as well as the first response group nSw-Vs (N1) and TGIP group mVs-Vs (M1) (**Figures 8B, C**). However, only seven DEGs, including Peroxidasin, Selenium-binding protein 1, were differentially expressed in the first response group nSw-Vs (N1) and TGIP group mVs-Vs (M1), may might be responsible for the enhanced immune response during maternal TGIP (**Figure 8C**). A total of 84 DEGs were assigned into groups related to the “signal transduction,” “infection disease,” “translation,” “cancers,” and “endocrine system.” In addition, upregulated genes appeared to be assigned to “immune system” and “cell communication” (**Figure 8D**).

**Figure 9:**
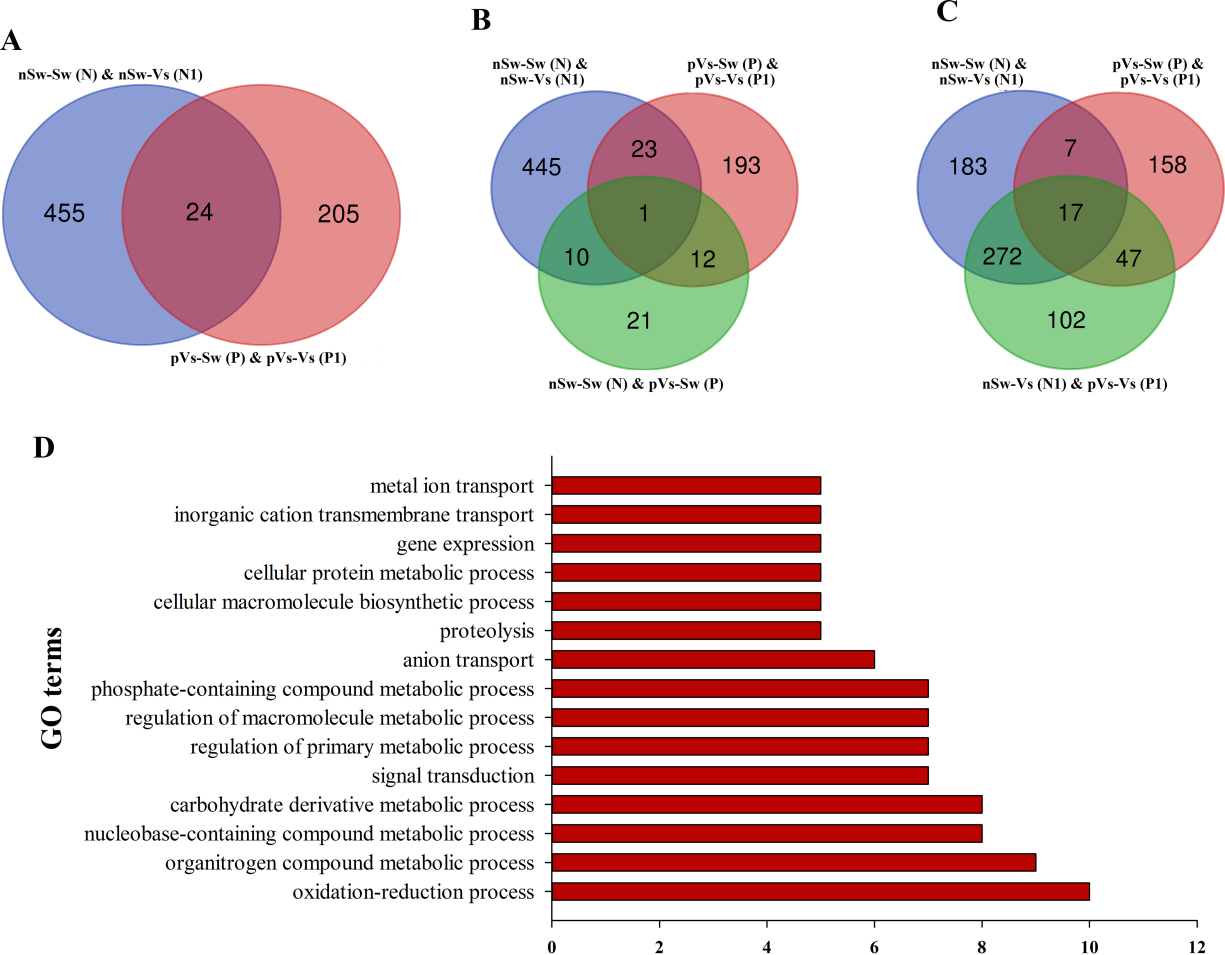
The analysis of differentially expressed genes in paternal primed larvae oysters. **(A)** The Venn diagram of differentially expressed genes in larvae oyster responding to both the first (against male oyster) and secondary stimulation of *V. splendidus*. **(B, C)** The Venn diagram of differentially expressed genes involved in paternal TGIP; **(D)** The GO enrichment of differentially expressed genes in oyster paternal TGIP.

A similar analysis was performed in the paternal TGIP, and 24 DEGs were screened by comparing nSw-Sw (N) and nSw-Vs (N1) with pVs-Sw (P) and pVs-Vs (P1) (**Figure 9A**). To explore DEGs between the unstimulated group (nSw-Sw (N)) and primed group pVs-Sw (P), as well as the first response group nSw-Vs (N1) and TGIP group pVs-Vs (P1) (**Figures 9B, C**), 17 DEGs (such as E3 ubiquitin–protein ligase NEDD1, laminin) were identified in the first response group nSw-Vs (N1) and TGIP group mVs-Vs (M1), which might be responsible for the enhanced immune response during maternal TGIP (**Figure 9C**). The GO term distributions of the 24 DEGs were analyzed, and the biological processes of the GO terms are shown in **Figure 9D**, showing that they were mainly distributed in the oxidation–reduction process.

### 3.9 The mRNA expression levels of *Cg*DNMT1, *Cg*DNMT2, and *Cg*DNMT3 during the oyster early ontogeny

**Figure 10:**
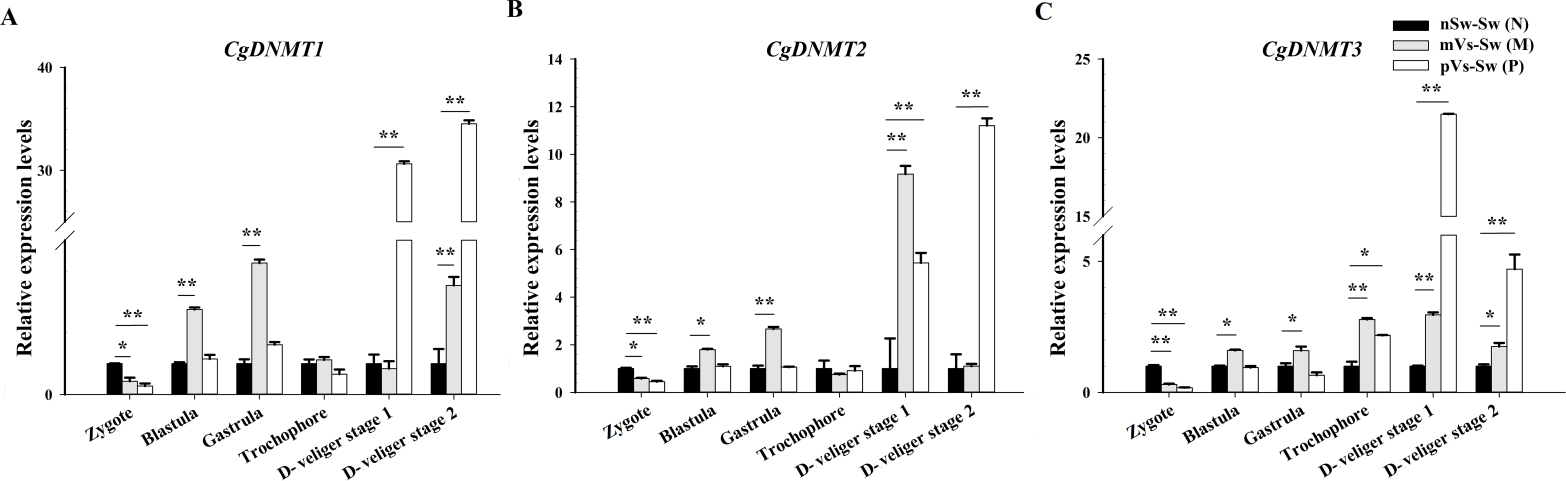
The mRNA expression levels of *CgDNMT1*, *CgDNMT2*, and *CgDNMT3* during the oyster early ontogeny. The relative mRNA expression levels of **(A)** *CgDNMT1*, **(B)** *CgDNMT2*, and **(C)** *CgDNMT3* in the control group nSw-Sw (N), maternal primed group mVs-Sw (M) and paternal primed groups pVs-Sw (P) at different developmental stages. Zygote (1 hpf), blastula (4.5 hpf), gastrula (8.5 hpf), trochophore larvae (15 hpf), D-veliger stage 1 (24 hpf), and D-veliger stage 2 (48 hpf). Vertical bars represent the mean ± SD (N≥3). *p <0.05, **p <0.01.

To investigate the potential regulation of DNA methylation in TGIP, the expression of three families of DNMTs was detected in the control group nSw-Sw (N), maternal primed group mVs-Sw (M), and paternal primed group pVs-Sw (P) at different developmental stages. In the maternally primed group mVs-Sw (M), the mRNA transcripts of the three DNMTs were low in the newly fertilized eggs (zygote, 1 hpf), but increased gradually to a relatively high level during oyster ontogeny (**Figure 10A**). Specially, the highest expressions of *CgDNMT1* appeared at the gastrula stage (8.5 hpf, *p <*0.01), and also higher at the blastula (4.5 hpf) and D-veliger stage 2 (48 hpf), which were 2.75- and 3.53-fold of that in the control group (*p <*0.05) (**Figure 10A**). The expression level of CgDNMT2 was highest at D-veliger stage 1 (24 hpf), which was 9.17-fold higher than that in the control group, while it was also higher at the gastrula stage (8.5 hpf, *p <*0.01) (**Figure 10B**). The mRNA transcripts of *CgDNMT3* increased gradually to a relatively higher level in the trochophore (15 hpf) and D-veliger stage 1 (24 hpf), which were 2.78- and 2.96-fold higher, respectively than those in the control group (*p <*0.01) (**Figure 10C**).

In the paternal primed group pVs-Sw (P), the expression patterns of the three DNMTs exhibited similar trends and showed lower or no significant difference during the early ontogeny (from zygote to gastrula) compared with the control group. In particular, the expression of *CgDNMT1* and *CgDNMT2* sharply increased after developing into D-veliger stage 1 (24 hpf), which was 30.64- and 5.44-fold higher than that in the in the control group, respectively (*p <*0.01), and reached their highest levels at D-veliger stage 2 (48 hpf), which were 30.64- and 5.44-fold higher than that in the in the control group, respectively (*p <*0.01) (**Figures 10A, B**). The expressions of *CgDNMT3* was significantly upregulated in trochophore larvae (15 hpf, 2.17-fold, *p <*0.05) and reached its highest level at D-veliger stage 1 (24 hpf, 21.50-fold, *p <*0.01) (**Figure 10C**).

### 3.10 The mRNA expression levels of E3 ligases during the oyster early ontogeny

**Figure 11:**
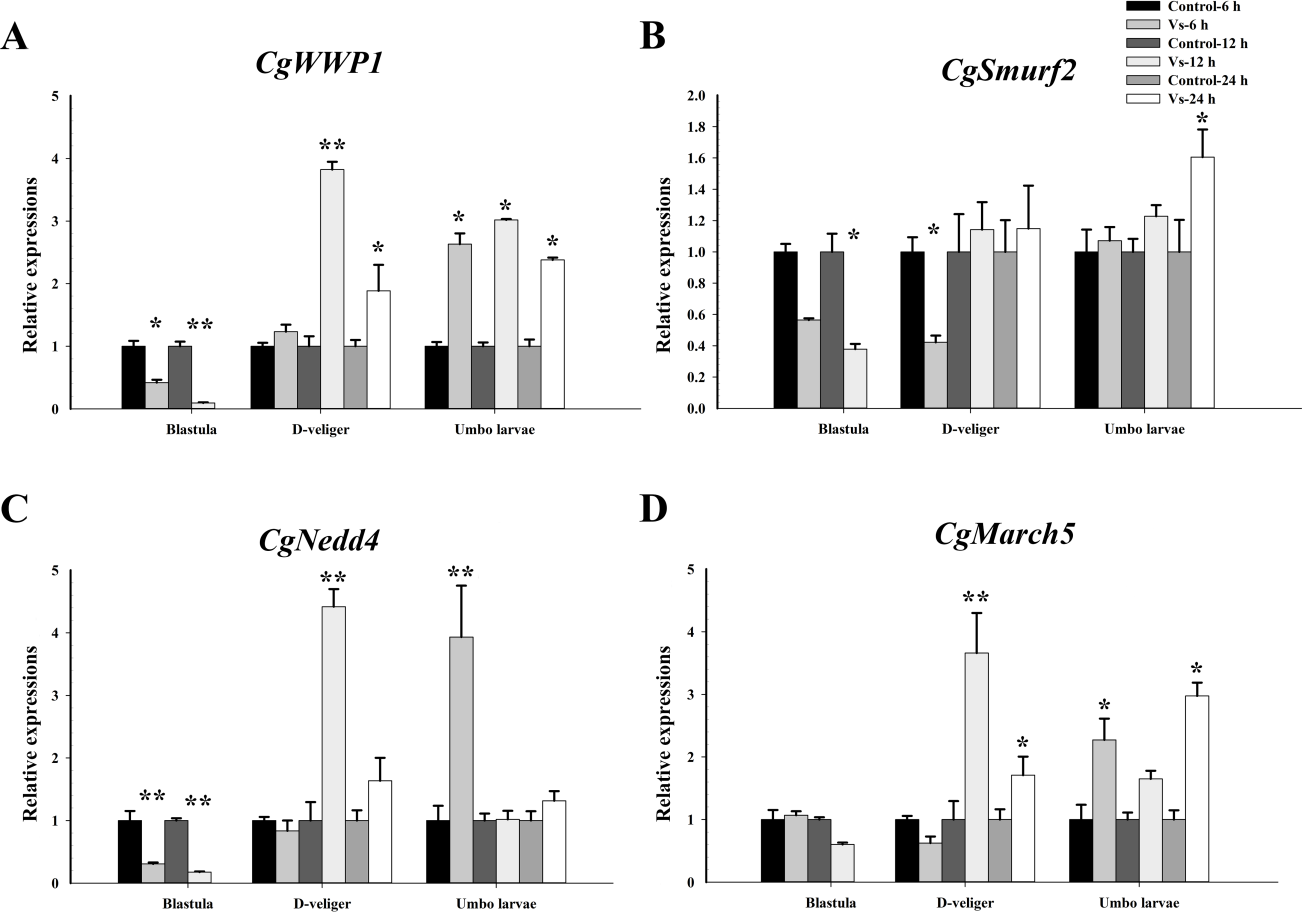
Temporal mRNA expression patterns of E3 ligases in *C. gigas* larvae after bacterial challenge. The relative mRNA expression levels of **(A)** *CgWWP1*, **(B)** *Cgsmurf2*, **(C)** *CgNedd4* and **(D)** *CgMarch5* at different time points post *V. splendidus* stimulation during the early ontogeny. Data was shown as mean ± S.E (n ≥6). *p <0.05, **p <0.01.

Based on transcriptome data, several E3 ligases were significantly induced after *V. splendidus* stimulation during ontogeny. Thus, to investigate the potential immune role of ubiquitination, the expression of four E3 ligases (*CgWWP1*, *Cgsmurf2*, *CgNedd4*, and *CgMarch5*) was examined at different time points after *V. splendidus* stimulation during early ontogeny. At the blastula stage (4.5 hpf), except for *CgMarch5*, the expression levels of the other three E3 ligases (*CgWWP1*, *Cgsmurf2*, and *CgNedd4*) were significantly downregulated at 6 h and 12 h post *V. splendidus* stimulation (*p <*0.05, **Figures 11A–C**). At D-veliger stage 1 (24 hpf), except for *Cgsmurf2*, the expression of the other three E3 ligases (*CgWWP1*, *CgNedd4*, and *CgMarch5*) was significantly upregulated at 12 h post *V. splendidus* stimulation, which were 3.82-, 4.42-, and 3.53-fold of that in the control group (*p <*0.01, **Figures 11A, C, D**). In the early umbo larvae (120 hpf), all four E3 ligases exhibited upregulation but distinct patterns after *V. splendidus* stimulation. Briefly, the mRNA transcripts of *CgWWP1* were significantly increased at 6 h, 12 h, and 24 h after *V. splendidus* stimulation (*p <*0.05, **Figure 11A**). The expressions of *CgMarch5* were upregulated at 6 h and 24 h after *V. splendidus* stimulation (*p <*0.05, **Figure 11D**), whereas the expression of *Cgsmurf2* and *CgNedd4* was upregulated at 6 h, and 24 h post *V. splendidus* stimulation, respectively (*p <*0.05, **Figures 11B, C**). These results indicate that E3 ligases might function in immunoregulation after the larvae develop into the D-veliger stage, when the immune system of oyster larvae has been developed to some extent.

## 4 Discussion

Oysters are constantly and repeatedly exposed to pathogens across generations, given that TGIP provides faster and stronger induction of oyster defense responses and enhanced resistance to biotic or abiotic stresses. It has been proposed that priming oyster offspring defense is a promising alternative approach in modern disease management (3). Although studies on TGIP have a long history in invertebrate systems, studies are yet to explicitly investigate and show evidence for bacteria-induced TGIP in mollusks.

Distinguished from the transfer of specific antibodies to their offspring in vertebrate females, innate immune factors are the main molecules transferred from the mother to offspring in invertebrates (51, 52). Previous studies have revealed that invertebrate eggs possess antibacterial and lysozyme activities as well as agglutination against pathogens (5). Several innate immune factors, including pattern recognition receptors (PRRs), such as lectins, and immune effectors, such as lysozyme, lipopolysaccharide binding protein/bacterial permeability-increasing proteins (LBP/BPI), and antioxidant enzymes, have been identified as maternally derived immune factors in molluscan eggs (51, 53, 54). Remarkably, increasing evidence has shown that maternal immune experience has a positive impact on the transfer of these immune factors, thus endowing offspring with enhanced immunity or disease resistance (30). For example, eggs from immune-challenged bumblebees exhibit greater antibacterial activity (9). This general pattern of elevated immunity has been described in a range of host taxa, such as insects, crustaceans, and nematodes, but has been less studied in mollusks. In the present study, immunity between egg extracts and hemocytes during the spawning season was detected by detailed temporal examination after *V. splendidus* stimulation. As expected, the expression of immunity-related genes encoding PRRs TLR2, as well as antibacterial proteins such as MACPF and FBG, was significantly induced in the hemocytes of *V. splendidus-*primed females and returned to resting time at 7 days post *V. splendidus* priming. Remarkably, immunity-related genes encoding antibacterial proteins, such as MACPF and FBG were also induced in the eggs of *V. splendidus-*primed females, and the upregulated trends lasted for one week until fertilization. Moreover, enhanced activities of lysozyme and antioxidant enzymes were detected in the eggs of *V. splendidus*, while no significant changes were observed in the hemocytes of *V. splendidus-*primed females. In addition, egg extracts from *V. splendidus-*primed females exhibited remarkably increased antibacterial activity. These results demonstrate the transfer of active innate immune components after maternal bacterial stimulation, which would endow mollusk eggs with effective defense against pathogen infection during ontogeny.

The phenomenon of invertebrate TGIP has received significant attention, although TGIP is not universal in invertebrates and is likely to depend on the host–pathogen system or the level of pathogen exposure (5, 9). For example, no evidence of TGIP was observed in the fruit fly *Drosophila melanogaster* after maternal challenge with as many as 10 different bacterial pathogens (55). Previous studies in our laboratory found that oysters exhibited a rapidly enhanced immune response after secondary *V. splendidus* stimulation, revealing that bacterial infection could trigger immune priming within generations (41). Other studies in oysters have found that exposure to diuron or various nucleic acids (such as poly (I:C)) during gametogenesis can prime oysters to trigger an anti-stress state and ultimately affect the offspring transcriptome, protecting them against subsequent stress (43, 56, 57). Thus, the present study directly measured immune parameters during the early life stages of maternal and paternal primed offspring. The results in the maternal-primed group suggested similar effects reported in insect taxa and vertebrates (3). For example, higher levels of enzymatic activity (e.g., CAT, lysozyme, and SOD) in primed offspring to induce immune-related genes (e.g., galectin, myd88, LBP), enhanced their survival and hatching upon bacterial challenge. Of particular note, boosting immunity, except for enzymatic activities, was also observed in the offspring originating from paternal primed oysters, supporting the view that paternally derived immune priming might also benefit from protecting their offspring (18, 32). Our data support that bacterial stimulation of the parental oyster can trigger an appropriate immune protein in offspring, as previously described for insects.

Currently, there are numerous reports concerning the molecular basis of TGIP mechanisms, and transcriptomics has been used as an initial step for comprehensive characterization of TGIP mechanisms in insect taxa (14). In this study, the priming of bacteria by female and male adults resulted in the differential expression of immunity-related genes in the offspring, providing evidence for TGIP in oysters. DEGs identified from the transcriptome in this study revealed that numerous immune factors as well as immune signaling pathways (e.g., TLR signaling pathway) were activated after parental bacterial priming, indicating that in addition to the direct transfer of active immune components, other mechanisms may be involved. It has been speculated that differences in immune function in offspring may be a result of transgenerational epigenetic changes caused by parental pathogen exposure (3, 37). Thus, DNA methylation levels were examined, and it was found that higher DNA methylation levels were detected at an earlier stage in the maternally primed group, but increased after development into the D-veliger larvae stage in the paternally primed group, which might be due to the different immune protection mechanisms originating from paternal and maternal factors. The Ubiquitin System is involved in the growth and development of organisms and in their response to biotic and abiotic stresses. Among these, E3 ubiquitin ligases play an important role in stress resistance by specifically recognizing substrates (58). Our previous study revealed the massive expansion of E3 ubiquitin ligase in transcriptomes under biotic and abiotic stresses (46, 50). In this study, several E3 ubiquitin ligases were significantly expressed upon larval exposure to bacteria, highlighting their potentially critical role in larval immunity.

## 5 Conclusion

In conclusion, we tested TGIP in the larval offspring of the oyster *C. gigas* after parental challenge with *V. splendidus*, and the results suggested that *V. splendidus* stimulation promoted the immunity tendentiously transferred to eggs during the spawning season. During early ontogeny, relatively lower mortality, higher hatching rates, and elevated immune effectors occurred when the larvae from primed parents re-encountered bacteria, confirming the existence of bacteria-triggered TGIP in oysters. Moreover, the results highlight the importance of not only considering the relative contribution of each parental sex to progeny performance, but also the combined effects that the two sexes may have on offspring performance. However, the present study focused mainly on the impact of TGIP on early life stages, and the persistent effects of TGIP during later ontogeny should be investigated in the future.

## Data Availability

The datasets presented in this study can be found in online repositories. The names of the repository/repositories and accession number(s) can be found below: BioProject ID: PRJNA1224195 (https://www.ncbi.nlm.nih.gov/search/all/?term=PRJNA1224195).
