# Supplementary figures and images for: Evidence for trans-generational immune priming against Vibrio splendidus in the oyster Crassostrea gigas
Source: Front Immunol. 2025 May 19;16:1536562. doi: 10.3389/fimmu.2025.1536562 (PMC12128116; doi:10.3389/fimmu.2025.1536562)

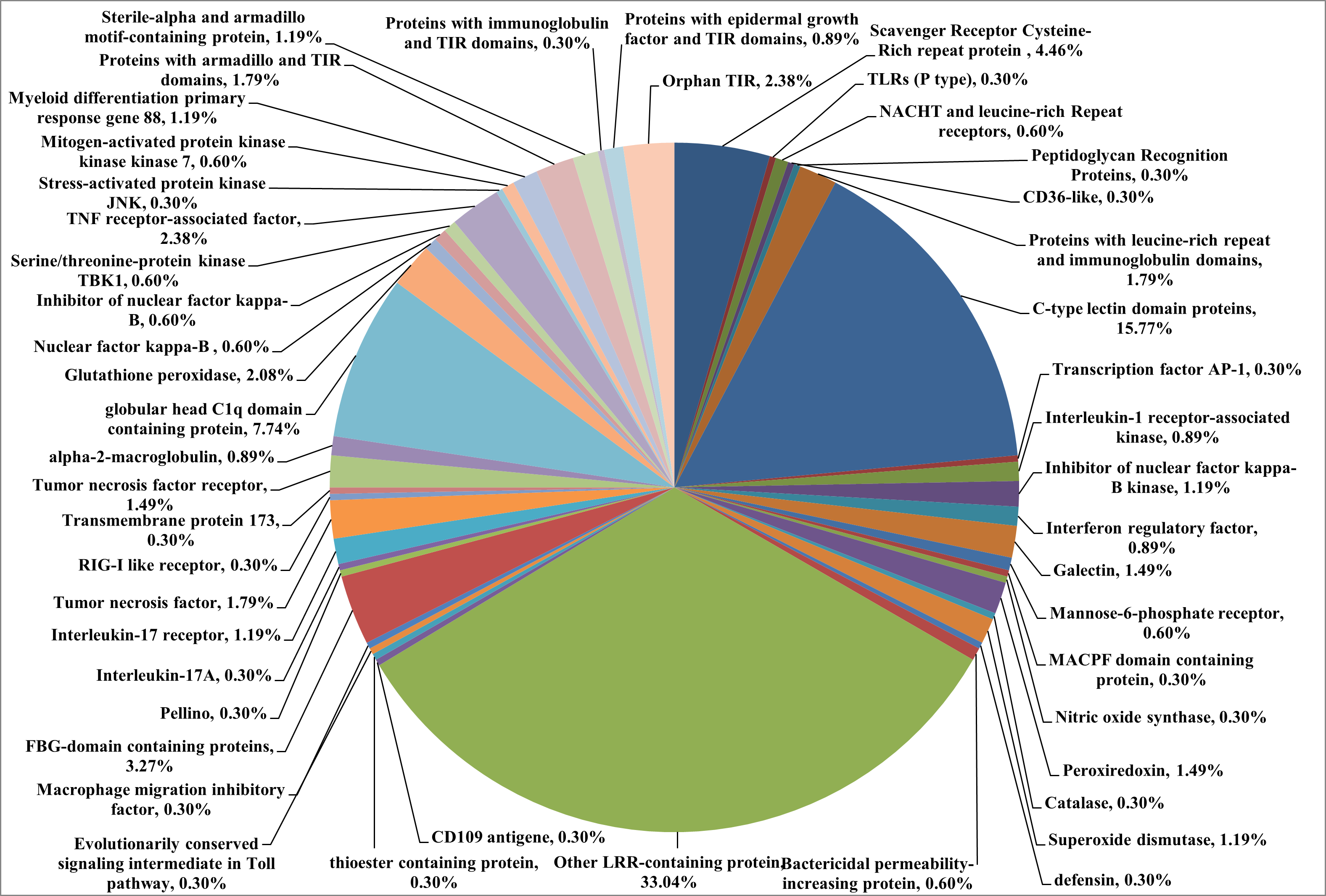

Supplement: Supplementary file 2 [file Image1.tif]
